# Evaluation of the Effect of Applying Chitosan, Neem, Tulsi, Aloe vera, and Chlorhexidine Solutions on the Shear Bond Strength of Composite to Dentin

**DOI:** 10.30476/dentjods.2023.98834.2112

**Published:** 2024-12-01

**Authors:** Farahnaz Sharafeddin, Fatemeh Aghaei

**Affiliations:** 1 Dept. of Operative Dentistry, Biomaterials Research Center, School of Dentistry, Shiraz University of Medical Sciences, Shiraz, Iran; 2 Postgraduate Student, Dept. of Operative Dentistry, Biomaterials Research Center, School of Dentistry, Shiraz University of Medical Sciences, Shiraz, Iran

**Keywords:** Resin composite, Chitosan, Neem, Tulsi, Aloe vera, Chlorhexidine, Shear bond strength

## Abstract

**Statement of the Problem:**

Dentin adhesion is challenging and needs modifications. Newly introduced nature-derived materials may be a useful solution in improving dentin adhesion. The use of natural antimicrobial agents for pretreating prepared dentin surfaces before restoration has become of interest.

**Purpose:**

The present study investigated the effect of natural compounds on the shear bond strength (SBS) of resin composite adhesively bonded to dentin.

**Materials and Method:**

Sixty extracted human molar teeth were randomly divided into six groups (n= 10); Group I: two-step etch and rinse adhesive system, Adper Single Bond 2 (SB_2_) (experimental control), Group II: 2.5% Chitosan+etch+SB_2_, Group III: 15% Neem (*Azadirachta indica*)+etch+SB_2_, Group IV: 15% Tulsi (*Ocimum sanctum*)+etch+SB_2_, Group V: 2% Aloe vera+etch+SB_2_, and group VI: 2% Chlorhexidine (CHX)+etch+SB_2_. Resin composite (Z350, 3M ESPE USA) was placed over the prepared dentin surfaces and was light cured. Data analysis was performed using One-way ANOVA and post hoc Tukey's test (*p*< 0.05).

**Results:**

Neem specimens showed the highest mean SBS, statistically greater than the control (*p*= 0.042). Other experimental groups showed no significant differences in SBS comparison to the control. The mean SBS values of 2.5% Chitosan, 15% Neem, 15% Tulsi, and 2% Aloe vera were significantly higher than 2% CHX (*p*= 0.046, *p*= 0.001, *p*= 0.010 and *p*= 0.002 respectively).

**Conclusion:**

Dentin pretreatment with Neem may improve the bond strength of a two-step etch and rinse adhesive system. The application of Chitosan, Tulsi, Aloe vera, and CHX did not demonstrate significantly different SBS values from that of the Control, although CHX was lower.

## Introduction

Resin-dentin bond stability is still a major concern in adhesive dentistry [ 1 ]. Destruction of the hybrid layer due to collagen fibrils degradation by matrix metalloproteinases (MMPs) and hydrolysis of resin, will compromise the bond strength [ 2 ]. Furthermore, the remained bacteria after tooth cavity preparation may deteriorate the microleakage and lead to secondary caries and pulpal response [ 1 ]. Thus in recent years, studies have focused on developing materials with eligible properties such as protease inhibitory effect, antibacterial activities, and remineralizing ability to promote resin-dentin bond longevity [ 3 - 6 ]. 

Disinfection of the prepared cavity after caries removal can aid in the elimination of microbial remnants [ 4 ]. Different materials have been proposed to improve dentin adhesion like Chlorhexidine digluconate (CHX), Neem (*Azadirachta indica*), and Aloe vera, before acid etching in tooth-colored restorations [ 4 ]. They act as antimicrobial agent matrix metalloproteinase (MMP) inhibitors, and so forth. MMPs and cysteine cathepsins, which are found in human dentin, contribute to the degradation of denuded collagen within the hybrid layer [ 2 ]. 

CHX is known as a gold standard cavity disinfectant in restorative dentistry [ 4 , 7 ] with antimicrobial activity [ 8 ], and MMPs inhibitory effect [ 9 ]. It has been shown that the degradation of dentin-bonds is reduced with the use of 2% CHX *in vivo* and *in vitro* [ 10 ]. However, recently, there has been a growing interest in investigating novel nature-derived materials for adhesive applications [ 11 - 12 ]. 

Chitosan, Neem, Tulsi (*Ocimum sanctum*), and Aloe vera are some new materials in dentistry that have favorable properties such as biocompatibility, biodegradability, non-toxicity, antibacterial activity, antioxidant properties, and anti-MMPs potential in addition to availability and low cost with no side effects [ 4 - 5 , 12 - 13 ]. 

Chitosan is a non-toxic biopolymer derived from alkaline deacetylation of chitin [ 14 ] and has increased the bond strength in comparison to dentin treated with phosphoric acid or untreated dentin in an *in vitro* study [ 15 ].

Neem is an evergreen tree [ 4 ] with a long history of being used to treat teeth and gum problems [ 16 ]. Aloe vera (Aloe barbadensis Miller) is an evergreen plant with antimicrobial activity [ 4 ] and anti-MMP properties [ 5 ] that may have several potential applications in dentistry [ 17 ]. It has been shown that Neem and Aloe vera have improved immediate and delayed SBS [ 4 ].

Tulsi is a holy plant of Indian origin [ 13 ] with antimicrobial activities against *streptococcus mutans* [ 18 ]. It has been shown that Tulsi leaf extract can reverse the negative effect of bleaching on resin-enamel bond strength [ 19 ].

Given that there is limited or no data about the effect of these new naturally occurring compounds on resin-dentin bond strength, and considering the need for comparative evaluation of newly introduced materials with each other and with traditional pretreatment materials, the purpose of this study was to investigate and compare the effect of Chitosan, Neem, Tulsi, Aloe vera and CHX on the SBS of resin composite to dentin. The research hypothesis was that pretreatment with these solutions would improve dentin bond strength and there would be no priority in improved SBS among tested groups.

## Materials and Method

The study was approved by the related Research Ethics Committee ​(IR.SUMS.DENTAL.REC.1399.024), sixty human third molar teeth with no sign of caries, cracks, defects, and restorations were collected, thoroughly cleaned, and kept in 0.1% thymol suspension at 4°C for 7 days. Afterward, the teeth were mounted in acrylic resin molds (Acropars, Iran) in a manner that the coronal portion of the teeth above the cement-enamel junction was left out of the mold and the occlusal surface was positioned in parallel with acrylic resin.

The occlusal surface of the teeth was sectioned with diamond discs (D and Z, Germany) under copious amounts of water, at a depth of 0.5 mm under the central pit. The sectioned surface was prepared with 600-grit silicon carbide paper (SiC paper, Piramit, Istanbul, Turkey) to obtain a uniform dentin surface. The teeth were randomly divided into six groups (n= 10) according to the pretreatment solutions. The commercial restorative products used in this study are listed in Table 1.

**Table 1:** Commercial restorative products of the study

Material	Manufacturer
Filtek^TM^ Z350 XT	3M ESPE, USA
Adper^TM^ Single Bond 2	3M ESPE, USA
Scotchbond^TM^ Etchant	3M ESPE, USA
Chitosan	Sigma Aldrich, USA
Self-cure acrylic resin	Acropars, Iran
LED polymerizing unit	Bluelex, GT 1200, Taiwan

Table 2 shows the description of dentin pretreatment materials of the study. 2.5% chitosan solution was prepared by adding 2.5 gr low molecular weight chitosan powder (Sigma Aldrich, USA) to 0.1% acetic acid. 15% Neem solution was achieved by adding 15 gr neem leaf powder (Indian Neem Tree Company, India) to 100 mL sterile distilled water, and filtration was done after 24 hours first using a silk cloth for coarse particles and followed by a Whatman no. 1 filter paper for fine residue [ 20 ]. 15% Tulsi solution (Herbal Heals, India) was prepared in the same manner. 2% Aloe vera solution prepared by adding 20 mg aloe vera powder (Nasim Faraz, Iran) to 100 ml sterile distilled water. As a 2% CHX solution, the Consepsis^TM^ (Ultradent, USA) solution was employed. The methods of dentin treatment are summarized in Table 3 and performed as follows.

**Table 2:** Description of dentin pretreatment materials

Material	Manufacturer	Description
2.5% Chitosan	Sigma Aldrich, USA	2.5 g of low molecular weight Chitosan was dissolved in 100 mL of 1% acetic acid
15% Neem	The Indian Neem Tree Company, India	15 gr of Neem leaf powder was added to 100 mL sterile distilled water and was filtered after 24 hours
15% Tulsi	Herbal Heals, India	15 gr of Tulsi leaf powder was added to 100 mL sterile distilled water and was filtered after 24 hours
2% Aloe vera	Nasim Faraz, Iran	20 mg Aloe vera powder (Nasim Faraz, Iran) to 100 ml sterile distilled water
2% Chlorhexidine	Consepsis, Ultradent, USA	2% Chlorhexidine gluconate solution

**Table 3:** Dentin pretreatments in the study

Groups	Treatment
Group 1	35% Phosphoric acid, 15s+SB_2_+resin composite
Group 2	2.5% Chitosan 30s+35% phosphoric acid, 15s+SB_2_+ resin composite
Group 3	15% Neem 30s+35% phosphoric acid, 15s+SB_2_+ resin composite
Group 4	15% Tulsi 30s +35% phosphoric acid, 15s + SB_2_ + resin composite
Group 5	2% Aloe vera 30s+ 35% phosphoric acid, 15s + SB_2_ + resin composite
Group 6	2% CHX 30s + 35% phosphoric acid, 15s + SB_2_ + resin composite

Adper Single Bond 2 (SB_2_), Chlorhexidine (CHX)

**Group 1:** (Control) 35% phosphoric acid (3M ESPE Scothbond Etchant, 3M ESPE, USA) was applied on the prepared dentin surface for 15 s and then rinsed for 10 s. After that, the dentin surface was blotted with a cotton pellet to remove excess water. Two coats of Adper Single Bond 2 (SB_2_) (3M ESPE, USA) were applied using a micro brush for 15 s immediately after blotting. Air thinning was then performed for 5 seconds to ensure solvent evaporation.

**Group 2:** Dentin was treated with 2.5% Chitosan solution using gentle rubbing motion of the micro brush for 30, then rinsed for 20 s with water [ 21 ] and dried using air spray.

**Group 3:** Dentin was treated with 15% Neem solution using gentle rubbing motion of the micro brush for 30s [ 4 ], then rinsed for 20s with water, and dried using air spray.

**Group 4:** Dentin was treated with 15% Tulsi solution using gentle rubbing motion of the micro brush for 30, then rinsed for 20 seconds with water, and dried using air spray.

**Group 5:** Dentin was treated with 2% Aloe vera solution using gentle rubbing motion of the micro brush for 30 [ 5 ], then rinsed for 20 s with water, and dried using air spray.

**Group 6:** Dentin was treated with an active application of 2% CHX solution (Consepsis, Ultradent, USA) with a micro brush for 30s [ 4 ], then rinsed for 20 s with water [ 22 ] and dried using air spray.

The treated and washed dentin surfaces were then acid etched (3M ESPE Scothbond Etchant) followed by the application of dentin bonding agent for groups 2 to 6 as was described in group 1.

**Figure 1:**
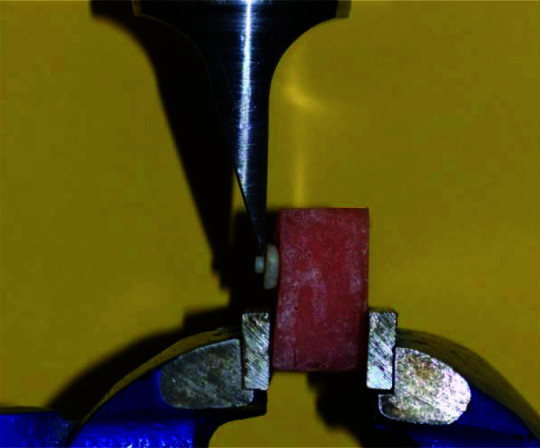
A sample in universal testing machine

The samples were light-cured for 10 s with a light emitting diode (LED) polymerizing unit (Monitex, Bluelex, GT 1200, Taiwan) at the light intensity of 1000 mW/cm2 and wavelength of 470 nm to cure the adhesive agent and light intensity evaluated by radiometer(DigiRate, Monitex, Taiwan). Resin composite (Filtek Z-250, 3M ESPE, USA) build-up was done by placing an uncured paste of resin composite into a 2 mm thick, 3 mm diameter hole of a clear plastic mold on the prepared dentin surface. The resin composite was then light-cured for 40s. The specimens were kept in distilled water for 24 h at 37°C in an incubator (ES 250 Nuve, Turkey). For shear bond testing, a shear load was to the upper side cylinder wall at the resin-dentin interface using a knife blade edge held in the crosshead of a universal testing machine (Zwick/Roell Z020, Germany) and lowered at a speed of 1.0 mm/minute (Figure 1).

The load observed at fracture (N) was divided by the cross-sectional specimen area to convert results into units of was calculated in MPa.

Statistical analysis was performed using SPSS soft ware, version 21 for Windows (SPSS Inc., Chicago, IL, USA). The Kolmogorov-Smirnov test assessed the normal distribution of the data (*p*< 0.05). One-way ANOV-A was implemented for data analysis, and multiple comparisons were done using post hoc Tukey's test (*p*< 0.05).

## Results

Mean SBS values and standard deviations (MPa) are presented in Table 4.

**Table 4:** Mean shear bond strength (MPa) values±SD in experimental groups

Groups	Treatments	Mean (MPa)± SD
Group 1	Control	12.53±1.866
Group 2	Chitosan 2.5%	13.62±1.816
Group 3	Neem 15%	15.30±2.276
Group 4	Tulsi 15%	14.15±1.500
Group 5	Aloe vera 0.2%	14.61±1.514
Group 6	CHX 2%	10.88±2.94

Chlorhexidine (CHX)

There was a normal distribution of the data in tested groups. One-way ANOVA showed a significant difference within the mean SBS values of experimental groups (*p*< 0.05). Pairwise means comparison of the experimental groups using Post hoc Tukey's tested for significant.

**Figure 2:**
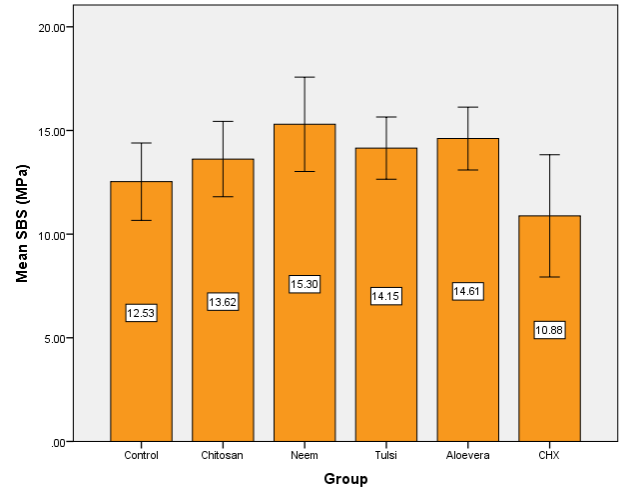
bar plot illustrates mean of the SBS (MPa) in all groups

The highest mean SBS value was observed with 15% Neem solution (Group 3) and the difference was statistically significant compared to the control (Figure 2). Dentin treatment with CHX 2% (Group 6) resulted in the lowest mean SBS value but was not significantly different from the control. Other experimental groups showed higher mean SBS values than the control without statistically significant differences. Groups 2 (Chitosan 2.5%), 3 (Neem 15%), 4 (Tulsi 15%), and 5 (Aloe vera 0.2%) led to significantly higher mean SBS values than group 6 (CHX 2%) (Table 5).

**Table 5:** Pairwise comparison of mean shear bond strength (SBS) values between experimental groups using Tukey's test

Groups (I-J)	Sig
1-2	0.840
1-3	0.042*
1-4	0.459
1-5	0.225
1-6	0.475
2-3	0.454
2-4	0.992
2-5	0.887
2-6	0.046*
3-4	0.878
3-5	0.954
3-6	0.000*
4-5	0.996
4-6	0.010*
5-6	0.002*

* The mean difference is significant at the 0.05 level

Group 1: Control, Group 2: Chitosan, Group 3: Neem, Group 4: Tulsi, Group 5: Aloe vera, Group 6: Chlorhexidine (CHX)

## Discussion

Recently there has been a trend to investigate natural biomaterials for dental applications, as the materials derived from nature have favorable properties, such as better biocompatibility, less toxicity, availability, and more affordable costs [ 12 , 23 ]. In this study, we investigated the SBS of resin composite to dentin with the application of different dentin pretreatment solutions such as Chitosan, Neem, Tulsi, Aloe vera, and CHX with anti-MMPs and antibacterial activity in an attempt to enhance the bond strength of resin composite to dentin. The SBS is widely used as a screen test for new adhesive materials [ 24 ], due to requiring minimum equipment, ease of specimen preparation and perform [ 25 ].

Different protocols have been reported for steps of application of phosphoric acid, treatment solution, and adhesive systems [ 25 - 27 ]. Chitosan, Neem, Tulsi, Aloe vera, titanium tetrafluoride, and CHX solutions have antimicrobial effects, so reducing microorganisms is an important factor in tooth-colored restorations before acid etching and dentin bonding application [ 4 , 13 , 27 ]. Therefore, in this *in vitro* study, we assessed new materials using a shear bond test before phosphoric acid gel.

According to the results of the present study, there was no significant difference between the mean SBS values of the experimental groups (Group 2,4-6) with the control group (Group 1) except for the samples pretreated with Neem solution (Group 3). So the null hypothesis was partially rejected.

Neem is a medicinal herb with numerous biological effects [ 28 ] such as antibacterial activity, especially against *Streptococcus mutans* and *Lactobacillus sp*, anti-MMPs activity against MMP-2 and MMP-9, and the ability to remove the smear layer [ 4 , 28 - 30 ]. In the present study, dentin pretreatment with Neem solution resulted in the highest mean SBS values amongst the tested groups and the difference was statistically significant compared to the control. Our finding is in agreement with the results of Shivika Goel *et al*. [ 4 ] study that revealed the application of Neem enhanced immediate and delayed bond strengths in comparison to the control. The positive effect of Neem on resin-dentin bond strength in our study may be attributed to its smear layer removal effect. On the other hand, we actively applied Neem solution on dentin, which may have enhanced the interaction of the material with the underlying dentin and helped in better smear layer removal resulting in higher bond strength to dentin.

One of the tested materials of the present study was Tulsi, which is considered a medicinal herb with potential promising applications in dentistry. To date, our study is the first research that evaluated the effect of Tulsi on resin-dentin bond strength. In the present study, Tulsi showed higher SBS than the control group but the difference was not significant. This finding may be due to its poor smear layer removal efficacy [ 28 ]. It should be noted that the most active chemical constituent of Tulsi is eugenol [ 31 ]. However, despite the known inhibitory effect of eugenol on the polymerization of methacrylate monomers and its incompatibility with the resin restorative systems [ 32 ], the bond strength was not compromised using Tulsi in the present study. It can be ascribed to the short exposure time of dentin to the eugenol-containing solution. Furthermore, 15% Tulsi solution was used which is low concentration, and dentin was not exposed to high amounts of eugenol. In addition, a two-step etch and rinse adhesive system was used, and the acid etch application after treatment of dentin with Tulsi may have removed eugenol and prevented interaction between eugenol and bonding agent. Since there is no similar study about the effect of Tulsi on the bond strength, it seems that the application of Tulsi as a cavity disinfectant may not compromise the bond strength of resin composite to dentin.

Aloe vera is a medicinal plant with desirable properties such as antibacterial, antioxidant, and anti-MMPs activity in addition to availability and biocompatibility [ 17 , 33 - 34 ]. In the present study, no significant increase in SBS was obtained with Aloe vera in comparison to the control. This finding is in contrast to the results of two recent studies conducted by Shivika Goel *et al*. [ 4 ] and Dakshina Joy Sinha *et al*. [ 5 ] that indicated Aloe vera improved the SBS using a two-step etch and rinse adhesive. Since the two studies mentioned above used the same adhesive system, and similar concentration and application time of Aloe vera to our study, the different yielded outcomes might be contributed to the step of application of the Aloe vera. They applied aloe vera after acid etching, while we applied Aloe vera before acid etching. Therefore, it might have been removed by etching and was not allowed to interact with dentin. Furthermore, Keerthana T *et al*. [ 35 ] showed that the application of aloe vera gel before a self-etch adhesive significantly reduces the SBS. This may be due to the application of Aloe vera in a high-viscosity gel form without following rinsing, which may have reduced contact area between the dentin surface and self-etch adhesive and inhibited the penetration of acidic monomers to the dentin surface. Consequently, the hybrid layer could not be formed properly resulting in decreased bond strength.

Chitosan is a natural biopolymer that has potentially beneficial properties in adhesive dentistry such as biocompatibility, a wide range of antibacterial activity, and the ability to improve the resistance of dentinal surface to degradation by collagenase [ 36 - 37 ]. Chitosan can remove or modify the smear layer depending on its concentration [ 3 ]. It also may play a role in opening interfibrillar spaces and may have a significant effect on resin permeation and the creation of a hybrid layer [ 21 ].

According to our findings, application of 2.5% Chitosan solution before acid etching of dentin resulted in higher mean SBS values without statistically significant difference to the control group. Similar to our finding, a study by Vasei *et al*. [ 14 ] showed that after 24 hours of water storage, Chitosan did not significantly affect the SBS. Obtaining no improvement in SBS may be due to following phosphoric acid application, which is strong enough to eliminate the smear layer. It can be hypothesized that the smear layer removal ability of 2.5% chitosan has no synergistic effect with phosphoric acid thus; it could not enhance the bond strength.

On the contrary, Lukram Nivedita *et al*. [ 21 ] showed that the application of Chitosan improved the SBS to dentin as compared to the control. It may be attributed to the step of Chitosan application, as we applied Chitosan before acid etching, but they applied Chitosan after acid etching of dentin. On the other hand, it has been shown that when the concentration of Chitosan increases, the bond strength value decreases [ 3 ]. In the study mentioned above, 1.2% Chitosan solution was used, while in the present study, we used 2.5% Chitosan solution. Therefore, different concentrations may have resulted in different outcomes.

In the current study, CHX showed lower SBS but was not significantly different from the control. This finding is in line with the outcomes of previous studies that showed CHX did not compromise the resin-dentin bond strength [ 10 , 38 - 39 ]. Contrary to our results, DW Elkassas *et al*. [ 40 ] found that pretreatment with CHX significantly reduced immediate bond strength when applied with a two-step etch and rinse adhesive. This contrast could be due to the different application times, as in their study CHX was applied for 60s, while we used CHX for 30s. It has been shown that CHX can stabilize the smear layer making it resistant to removal by etchant [ 38 ]. It seems that the stabilizing effect of CHX on the smear layer may compromise the bond strength in prolonged application of CHX.

In the present study, CHX showed significantly lower bond strength compared to other experimental materials. The superiority of Chitosan, Neem, Tulsi, and Aloe vera to CHX in terms of bond strength suggests that these materials may be good alternatives to CHX as a cavity disinfectant. However, more research is needed to validate this laboratory finding before the presentation of these materials for widespread use in adhesive dentistry.

This *in vitro* study was performed in laboratory conditions, which might not simulate the oral environment variables completely. Thus, future *in vivo* studies are suggested before the generalization of the outcomes to clinical applications. In addition, further studies are recommended to investigate the long-term effect of tested materials on the SBS, other application methods, and other adhesive systems.

## Conclusion

Based upon the limitations imposed by the methods of the current study, it can be concluded that dentin pretreatment with Neem may enhance bond strength of resin composite to dentin. Dentin pretreatment with Chitosan, Tulsi, Aloe vera, and CHX did not compromise resin-dentin bond strength. Higher bond strength was obtained with Chitosan, Neem, Tulsi, and Aloe vera in comparison to CHX. Hence, these materials might be good alternatives to CHX as a cavity disinfectant.
